# IgA autoimmunity and coagulation among post-acute sequelae of SARS-CoV-2 infection (PASC) patients with persistent respiratory symptoms: a case-control study

**DOI:** 10.3389/fimmu.2025.1589559

**Published:** 2025-05-30

**Authors:** Claudia Gomes, Jonathan H. Whiteson, Fabio Ponzo, Rany Condos, Mila B. Ortigoza, Marisol Zuniga, Ana Rodriguez, David C. Lee

**Affiliations:** ^1^ Department of Microbiology, NYU Grossman School of Medicine, New York, NY, United States; ^2^ Department of Rehabilitation Medicine, NYU Grossman School of Medicine, New York, NY, United States; ^3^ Department of Medicine, NYU Grossman School of Medicine, New York, NY, United States; ^4^ Division of Nuclear Medicine, Department of Radiology, NYU Grossman School of Medicine, New York, NY, United States; ^5^ Division of Pulmonary, Critical Care, and Sleep Medicine, Department of Medicine, NYU Grossman School of Medicine, New York, NY, United States; ^6^ Division of Infectious Diseases, Department of Medicine, NYU Grossman School of Medicine, New York, NY, United States; ^7^ Ronald O. Perelman Department of Emergency Medicine, NYU Grossman School of Medicine, New York, NY, United States; ^8^ Department of Population Health, NYU Grossman School of Medicine, New York, NY, United States

**Keywords:** PASC, respiratory distress, microvascular thrombosis, nuclear medicine, SPECT-CT, thromboelastography, autoimmunity

## Abstract

**Introduction:**

The SARS-CoV-2 virus resulted in significant disability and diagnostic challenges among patients with Post-Acute Sequelae of COVID-19 (PASC). Here, we assessed microvascular perfusion, clotting, and autoimmune responses to lung targets in PASC patients compared to healthy controls with the aim of explaining the persistent respiratory symptoms of patients with PASC.

**Methods:**

We performed a blinded case-control study of 20 PASC patients with persistent respiratory symptoms versus 20 healthy controls previously infected with SARS-CoV-2 virus. We assessed lung perfusion using Technetium-99m macroaggregated albumin (MAA) SPECT-CT scans, clotting using coagulation and thromboelastrogram (TEG) tests, and autoimmunity to vascular and lung antigens using ELISA assays.

**Results:**

Subjective respiratory symptoms and quality-of-life measures were significantly worse among the PASC patients compared with healthy controls (p<0.001). Clinical symptoms among PASC patients were inversely correlated with plasma total IgA levels (coefficient: -0.61, p=0.004) and with autoimmune IgA recognizing pulmonary microvascular endothelial cell antigens (coefficient: -0.51, p=0.02). Additionally, levels of total IgA were directly correlated with fibrinogen and fibrin-related clot strength (coefficient: +0.52, p=0.02; coefficient: +0.63, p=0.003). SPECT-CT scans were positive only among 25% of PASC cases versus 10% of healthy controls (p=0.41). TEG tests showed no differences between the groups.

**Conclusions:**

Our small study of PASC patients identified that circulating IgA antibodies may correlate inversely with clinical symptoms and directly with clotting parameters, suggesting a possible link between autoimmunity and coagulation. However, many of the study’s findings were null, which may mean that tissue-level studies or alternative explanations of PASC need to be explored.

## Introduction

The underlying pathophysiology of Post-Acute Sequelae of COVID-19 (PASC) has yet to be clarified, however, several leading theories have been proposed. These include the lingering effects of direct damage caused by the SARS-CoV-2 virus, possible persistence of the virus in certain organs and tissues, dysregulation of the immune system with ongoing inflammation and/or a loss of self-tolerance resulting in autoimmunity (1). Though the exact causes of have not been established, there is emerging evidence suggesting that PASC patients may be experiencing a more chronic form of the prothrombotic state that is known to occur after SARS-CoV-2 infection (2).

Advanced nuclear medicine scans have revealed that some PASC patients may have perfusion deficits at a microvascular level (3, 4), which is consistent with pathologic findings described in autopsies among fatal cases of acute COVID-19 (5, 6). Evidence for microvascular thrombosis has been suggested by recent studies of abnormal microclots that have been identified in the plasma of PASC patients, which may be induced by abnormal platelet activation and a dysfunction of fibrinolysis (7, 8).

The underlying pathophysiology that would drive such possible disturbances in clotting mechanisms is unclear. However, there have been many short- and long-term complications of SARS-CoV-2 infection that strongly suggest autoimmune involvement (9, 10). Furthermore, autoimmunity is thought to be one of the more likely causes of PASC with identification of various autoantibodies which appear to be persistent (11). However, a specific autoantigen target that is recognized by a majority of patients with persistent debilitating symptoms after SARS-CoV-2 infection has not be identified. In the years following the COVID-19 pandemic, there also have been several studies suggesting that abnormal clotting and autoimmunity might driving the pathophysiology of PASC, but most of the analyses have been theoretical rather than an empirical study of PASC cases versus healthy controls (12–15).

In this study, our first aim was to assess the clinical utility of using thromboelastography (TEG) and lung perfusion via Single Photon Emission Computed Tomography scan with a Computed Tomography scan (SPECT-CT) scans to assess clotting abnormalities in PASC patients with persistent respiratory symptoms versus healthy controls with prior SARS-CoV-2 infection. In the same cohort, our second aim was to determine the levels of plasma autoimmune IgG and IgA antibodies to study possible associations between autoimmunity, coagulation and lung pathophysiology as we hypothesized these factors may be driving the persistent shortness of breath experienced by some PASC patients.

## Materials and methods

### Study design

We performed a case-control study to identify clotting abnormalities, microvascular perfusion deficits, and lung autoimmunity among PASC patients with persistent respiratory symptoms versus healthy controls with prior SARS-CoV-2 infection. In addition to a medical history, two validated questionnaires, and laboratory tests, each patient received a perfusion SPECT-CT of the lung. The nuclear medicine specialist reading the scans was blinded to the status of study participants as cases or controls and did not review the charts of study participants. Plasma samples were also collected to study autoimmunity to vascular and lung antigens. The NYU School of Medicine IRB approved our study protocol s21-01523.

### Study population

Cases in the study cohort were recruited from the PASC Clinics at NYU Langone Health. To be eligible, cases had to meet the clinical case definition of post-COVID-19 syndrome as determined by the World Health Organization and have persistent respiratory symptoms without alternative diagnosis (e.g., shortness of breath, dyspnea on exertion). We excluded cases with significant parenchymal lung disease (e.g., pulmonary fibrosis, ground glass opacities), which might otherwise explain the presence of ongoing respiratory symptoms among these PASC patients.

Healthy controls were selected among individuals who had a prior SARS-CoV-2 infection but had complete resolution of symptoms at the time of enrollment. For this study, cases and controls had to have documented evidence of prior SARS-CoV-2 infection with either a positive PCR, antigen, or pre-vaccination anti-spike antibody result. However, it is important to note that not all participants had access to testing at onset of their initial infection. In four cases, testing was not available during their initial infection, but two of these individuals tested positive during a second SARS-CoV-2 infection prior to enrollment. We also excluded individuals who were pregnant, breastfeeding, or had a history of pulmonary embolism or chronic kidney disease or had an allergy to intravenous contrast or nuclear medicine tracer compounds. Healthy controls were offered a $50 incentive for their participation in the study.

### Data collection

All patients provided a detailed history of their prior SARS-CoV-2 infection including dates, test results, persistent and resolved symptoms. In addition, they answered questions about their past medical history and responded to two validated surveys: the St. George’s Respiratory Questionnaire (SGRQ), which has been validated for chronic obstructive pulmonary disease, asthma, and several other respiratory diseases (16), and the RAND 36-Item Short Form Survey, which provides an assessment of quality-of-life including physical health and mental health scores.

### Laboratory tests

Prior to the nuclear medicine scans, study participants also had blood tests to check for renal dysfunction (creatinine) and pregnancy (beta hCG among women). In addition, we collected coagulation profiles (PT, INR, aPTT, thrombin time, fibrinogen), d-dimer, and thromboelastography (TEG) with clot lysis and platelet mapping. These blood tests were performed using standardized assays and reporting values by the clinical laboratory at the Manhattan campus of NYU Langone Health.

### Nuclear medicine imaging

Study participants underwent a SPECT-CT of the lung, which included an initial CT without contrast that evaluated for any structural pathology and provided a scaffold for the nuclear medicine scan. Patients then received an intravenous injection of radiopharmaceutical Tc-99m macroaggregated albumin (MAA) to the lung perfusion portion of the SPECT-CT study. The nuclear medicine specialist remained fully blinded to the status of participants as cases or controls until completion of the study.

### Quantification of plasma antibody levels and total cell-free DNA

Patient plasma samples were obtained and stored at -80°C during ongoing recruitment of study participants. Total levels of IgG, IgA, Immune-complexes and reactivity against anti-SARS-CoV-2 spike protein, two lung cell lines (human pulmonary microvascular endothelial cells - HPMEC (17) and human lung adenocarcinoma epithelial cells A549 (ATCC)), DNA, Annexin A2 and A5 was determined by ELISA in relative units (RU) as compared to a positive control autoantibody sample. Specific protocols for each ELISA can be found in the **Supplementary Methods** section. Total cell free DNA was measured in 1µL plasma using the dsDNA HS assay kit from Invitrogen and a Qubit 2.0 fluorometer, following the manufacturer’s instructions.

### Statistics

We first described our study population comparing cases versus controls by age, sex, race/ethnicity, past medical history, time since first infection, vaccination status, post-COVID-19 symptoms, SF-36 scores, SGRQ scores, and laboratory results. Categorical variables were compared using Fisher’s exact test given the low sample size, and continuous variables were compared using t-tests in order to compare continuous factors between the two groups. Our primary outcome of microvascular thrombosis on SPECT-CT scan was compared between cases and controls using Fisher’s exact tests given the low sample size. To test for differences in IgG and IgA antibody activity against selected targets, IgG and IgA immune complexes, and total plasma IgG and IgA levels, we used Mann-Whitney given the non-normal skew in the resulting values and plotted results using box and whisker plots. Pairwise Pearson correlations tests were performed between clinical symptom scores, coagulation lab tests, and thromboelastrography results with antibody levels identified by ELISA to identify statistically significant associations.

Statistical analyses were performed using Stata 16.1 (StataCorp; College Station, TX, 2019) or GraphPad Prism 9.5.1 (GraphPad Software; Boston, MA). SGRQ scores were calculated using the SGRQ App v.3.0.12 (Copenhagen University Hospital; Denmark).

## Results

### Study population

Comparing the 20 cases of PASC patients with persistent respiratory distress versus the 20 healthy controls previously infected with SARS-CoV-2 virus, the demographic characteristics in terms of age, sex, race and ethnicity were similar (**Table 1**). As for medical history, there were no statistically significant differences in past medical history. However, there were a higher proportion of cases who reported a history of irritable bowel syndrome and anxiety prior to COVID-19. There was a statistically significant difference between time since first SARS-CoV-2 infection among cases, which was a median of 24 months compared to controls which was 7 months (p-value = 0.01). In terms of vaccination status, there was one case that was unvaccinated, however, the vaccine was not available prior to the first infection of each case except for two cases who developed PASC symptoms after being fully vaccinated (**Table 1**). Notably, no cases or controls had severe COVID-19 as no study participant required hospitalization due to their SARS-CoV-2 infection. (See **Supplementary Figure S1** for flow diagram of study participant recruitment.)

**Table 1:** Study population comparing PASC cases with persistent respiratory symptoms versus healthy controls after SARS-CoV-2 infection.

Characteristics	Healthy Controls After SARS-CoV-2 Infection	Respiratory PASC Cases	Significance
*Age*			
Median	33	42	0.06
Range	26 to 62	25 to 67	
*Sex*			
Male	50%	25%	0.09
Female	50%	75%	
*Race/Ethnicity*			
White	60%	55%	0.91
Black	5%	15%	
Hispanic	25%	20%	
Asian	10%	10%	
*Medical History Prior to SARS-CoV-2 Infection*			
Irritable Bowel Syndrome	5%	25%	0.09
**Anxiety**	**15%**	**50%**	**0.04**
*Months Since First Infection*			
**Median**	**7**	**24**	**0.01**
Range	3 to 26	4 to 29	
*Vaccination Status*			
Not Vaccinated	0%	5%	1.00
**Vaccine Not Available** **Prior First Infection**	**35%**	**75%**	**0.03**
*Post-COVID-19 Symptoms*			
**Respiratory**	**0%**	**100%**	**< 0.001**
**Cardiovascular**	**0%**	**100%**	**< 0.001**
**Neurologic**	**0%**	**90%**	**< 0.001**
*RAND SF-36 Scores*			
Physical Health Score			
**Average**	**56**	**37**	**< 0.001**
Range	46 to 63	17 to 53	
Mental Health Score			
**Average**	**53**	**36**	**< 0.001**
Range	31 to 60	17 to 59	
*St. George’s Respiratory Questionnaire Scores*			
**Total Scores Average**	**1**	**52**	**< 0.001**
Range	0 to 3	19 to 76	
**Symptoms Average**	**5**	**51**	**< 0.001**
Range	0 to 21	24 to 83	
**Activity Average**	**1**	**66**	**< 0.001**
Range	0 to 6	6 to 100	
**Impact Average**	**0**	**43**	**< 0.001**
Range	0 to 0	11 to 66	

Significant differences highlighted in bold.

### Persistent symptoms and questionnaires

All cases reported persistent respiratory symptoms (e.g., shortness of breath, difficulty breathing on exertion, or hypoxia). In addition, all cases also reported persistent cardiovascular symptoms (e.g., dizziness, lightheadedness, palpitations, or changes in blood pressure upon standing). All but two cases also reported cognitive dysfunction, and after enrollment, it was identified that one of the healthy controls did report nine months of cognitive issues after their SARS-CoV-2 infection, but these symptoms had resolved for the past 12 months prior to enrollment. Based on SF-36 scores, cases had significantly lower average physical health scores of 35 compared to controls with an average of 56 (p-value < 0.001). Average mental health scores were also lower among cases with an average of 35 compared to controls with an average of 53 (p-value < 0.001). In addition, the SGRQ scores differed significantly between the two groups with cases having an average of 51 out of 100, compared to controls who all essentially reported minimal or no respiratory symptoms with scores between 0 and 3 (p-value < 0.001).

### Laboratory and imaging results

Aside from a small difference between measured thrombin times (20.8 among cases versus 19.9 among controls), there were no significant differences between study groups in terms of creatinine, platelet count, and coagulation profiles, which included fibrinogen and d-dimer. Notably, only two study participants had an elevated d-dimer results, which was one case and one healthy control. No statistically significant differences were noted on standard TEG parameters nor platelet mapping parameters. Cell-free DNA showed a significant decrease in the PASC cases group (**Table 2**).

**Table 2:** Lab and imaging results among PASC cases with persistent respiratory symptoms versus healthy controls after SARS-CoV-2 infection.

Average Lab Values and Imaging Results	Healthy Controls After SARS-CoV-2 Infection^1^ n = 20	Respiratory PASC Cases^1^ n = 20	Significance^2^ p-values
*Coagulation Profile / Cellular damage*			
PT (sec)	11.1	11.0	0.71
INR (ratio)	0.99	0.98	0.64
aPTT (sec)	34.6	34.6	0.96
**Thrombin Time (sec)**	**19.9**	**20.8**	**0.04**
Fibrinogen (mg/dL)	306	309	0.90
D-dimer (ng/mL)	122	133	0.54
**Cell-free DNA (ng/μL)**	**1.40**	**1.19**	**0.04**
*Thromboelastography*			
Reaction Time (min)	6.2	6.0	0.56
Clot Lysis (in 30 mins)^3^	1.0%	1.4%	0.20
Max Amplitude (MA) (millimeters)	59.2	59.0	0.91
MA – Fibrinogen Function (mm)	19.2	20.2	0.28
*Platelet Mapping*			
MA – Kaolin with Heparinase (mm)	60.0	59.5	0.65
MA – Adenosine Diphosphate (mm)	53.1	55.5	0.42
MA – Arachidonic Acid (mm)	51.1	45.8	0.29
MA – Activator F (mm) (Factor XIII)	9.0	9.4	0.75
*SPECT-CT Scan*			
% with Perfusion Deficits on SPECT	10	25	0.41
% with Airway Disease on Non-contrast CT	0	10	0.49

^1^Values represent the average of all determinations in each group.

^2^Significance was calculated using unpaired *t*-test.

^3^Missing one case that did not have a clot lysis analysis performed. Bold values were statistically significant.

**Figure 1:**
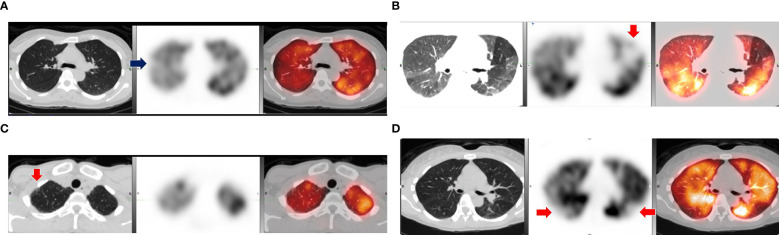
Comparison of abnormal lung SPECT-CT results among cases and controls with positive findings. **(A)** Small wedge-shaped perfusion defect in lateral segment of right upper lobe (arrow). **(B)** Medium size defect in anterior left upper lobe (arrow) and evidence of air trapping. **(C)** Small airway disease in anterior right upper lobe (arrow) without perfusion defect. **(D)** Control with multiple subsegmental perfusion defects in bilateral lower lobes (arrows). Notes: Scans of positive cases **(A–C)** and a positive control **(D)** with CT scan (left), SPECT perfusion scan (middle), and fused SPECT-CT (right).

As for SPECT-CT results, 5 of the 20 (25%) PASC cases scanned had evidence of microvascular perfusion deficits versus 2 of the 20 (10%) healthy controls (p-value = 0.41). Among the cases, four abnormalities were described as small subsegmental perfusion deficits in a segment of a single lobe on one side of the lung and one of the abnormalities was described as multiple small to large deficits in various segments of both lungs. Among the controls, one abnormality was described as a small subsegmental perfusion deficit in a segment of a single lobe on one side of the lung, and the other was described as multiple small to large deficits in various segments of both lungs. In addition, it was incidentally noted that 2 of the 20 (10%) PASC cases had evidence of airway disease, one being evidence of air trapping matching the perfusion deficits seen for the case that had bilateral lung perfusion deficits and another case that had evidence of small airway disease in the anterior upper lobes bilaterally without any evidence of perfusion deficits (**Figure 1**). Each series of images demonstrates the lung architecture from the CT scan; followed by the perfusion imaging, which demonstrates areas of blood flow; and a resulting combined image, which overlays the perfusion in color on top of the structure of the lung.

### Levels of circulating antibodies

**Figure 2:**
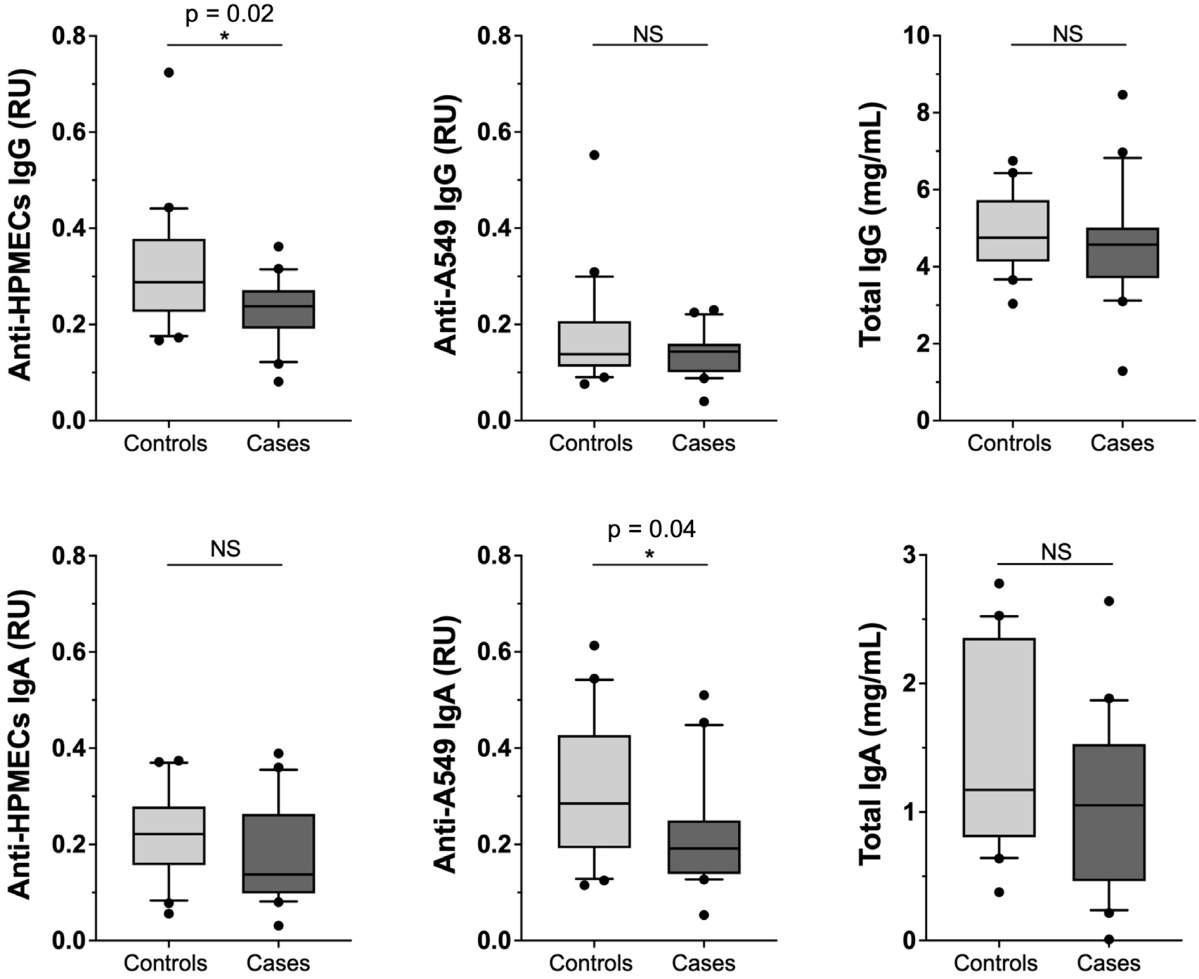
Comparison among PASC patients versus healthy controls of total IgG and IgA antibody levels and of autoimmune antibody levels against lung endothelial and epithelial cell lines. Levels of anti-HPMEC and anti-A549 IgG and IgA along with Total Plasma IgG and IgA from cases (n = 20) or controls (n = 20). RU (Relative Units). Significance assessed by unpaired Mann-Whitney test *p < 0.05.

To explore the possibility of autoimmunity, we analyzed plasma samples for evidence of IgG and IgA antibodies against lung endothelial (HPMEC) and epithelial (A549) cell lines (**Figure 2**). While the levels of total IgG and IgA antibodies did not differ in PASC patients and controls, both IgG and IgA anti-endothelial cell antibodies (i.e., binding in ELISA plates coated with HPMEC lysates) were lower among cases compared to controls, but only met statistical significance for IgG (p-value = 0.02). As for anti-epithelial antibodies, only the IgA antibodies were lower among PASC cases versus controls (p-value = 0.04). The levels of circulating IgG and IgA Immune complexes or specific autoimmune antibodies, such as anti-DNA, anti-annexin-A2 and anti-annexin-A5 did not show differences between PASC patients and controls (**Supplementary Figure S2**) (10).

### Correlational analysis of clinical symptoms, laboratory tests results and circulating antibody levels in PASC patients

**Figure 3:**
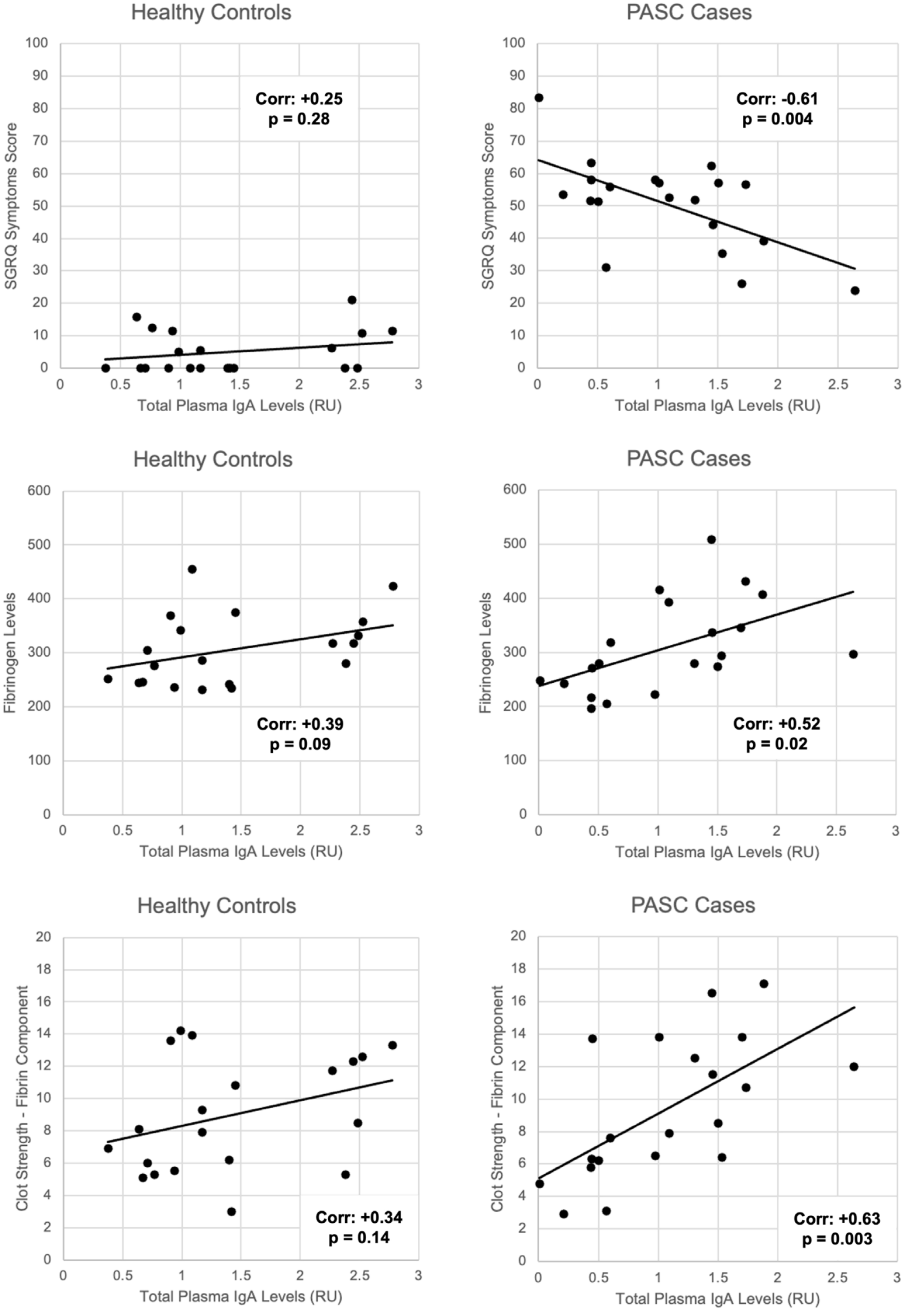
Correlations between total plasma IgA levels and SGRQ symptoms, fibrinogen levels, and fibrin-related clot strength among PASC patients versus healthy controls. Linear correlations depicted by black lines. Corresponding Pearson correlation coefficients (corr.) and p-values for significance labelled on each graph.

**Figure 4:**
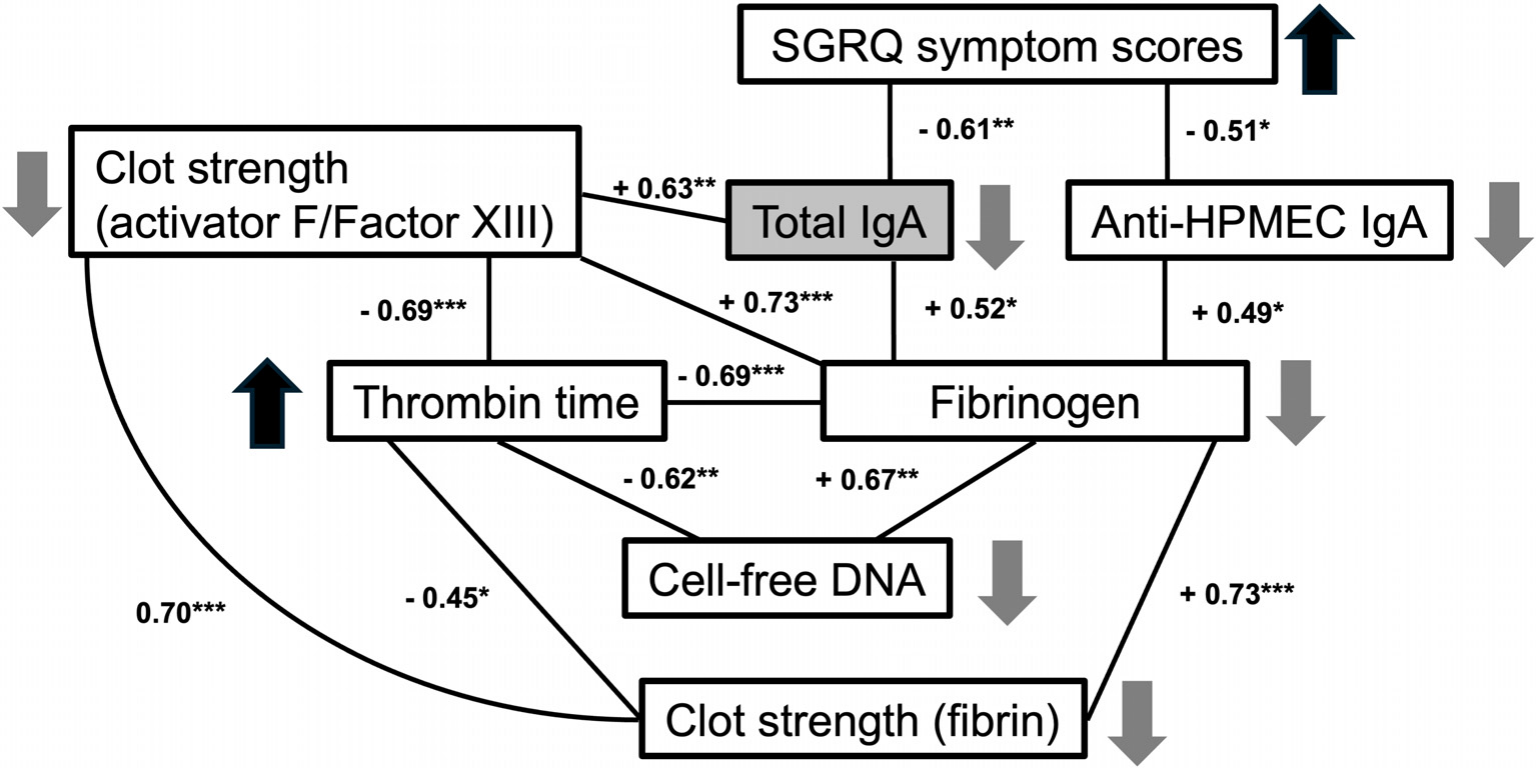
Correlations between symptoms, IgA and coagulation parameters in PASC patients. Numbers indicate correlation Pearson coefficient between each two parameters (*p < 0.05, **p < 0.01, ***p < 0.001). Upward and downward pointing arrows indicate an increase or decrease of each parameter related to decreased total IgA levels (as found in PASC patients with increased symptomatology).

We identified that the levels of total plasma IgA, and in particular of anti-endothelial IgA antibodies (anti-HPMEC), in PASC patients correlate inversely with respiratory symptoms. Additionally, total IgA was correlated with coagulation parameters: directly with fibrinogen and clot strength (TEG activator F; Factor XIII) (**Figures 3**, **4**). These statistical relationships were not observed among healthy controls and support the study’s main hypothesis that autoimmunity and clotting may be inter-related and could suggest an underlying pathophysiology of the persistent respiratory symptoms of PASC.

We also observed that certain coagulation-related parameters, such as thrombin time and fibrinogen were strongly associated with cell-free DNA and with factors contributing to clot strength as determined by TEG, such as fibrin and activator F (Factor XIII) (**Figure 4**). Other autoimmune IgA antibodies, including anti-epithelial, anti-DNA, anti-annexin A2 and anti-Annexin A5 antibodies, were directly associated with prothrombin time in PASC patients (**Table 3**). Taken together, these findings suggest a possible relationship between coagulation and autoimmunity among PASC patients with persistent respiratory symptoms.

**Table 3:** Correlations between autoimmune antibodies and prothrombin time among PASC cases with persistent respiratory symptoms and healthy controls after SARS-CoV-2 infection.

Antibodies Tested	Prothrombin Time			
	Healthy Controls (n=20)		PASC Cases (n=20)	
	Correlation^1^	Significance^2^	Correlation^1^	Significance^2^
Total IgG	-0.137	0.57	0.214	0.36
Total IgA	0.114	0.63	0.377	0.10
Anti-A549 IgG	0.128	0.59	0.287	0.22
**Anti-A549 IgA**	0.151	0.53	**0.574**	**0.01**
Anti-HPMEC IgG	-0.075	0.75	0.361	0.12
Anti-HPMEC IgA	0.163	0.49	0.274	0.24
Anti-DNA IgG	0.108	0.65	0.283	0.23
**Anti-DNA IgA**	0.364	0.12	**0.523**	**0.02**
Anti-AnnexinA2 IgG	0.049	0.84	0.255	0.28
Anti-AnnexinA2 IgA	0.063	0.79	0.411	0.07
Anti-AnnexinA5 IgG	0.038	0.87	0.254	0.28
**Anti-AnnexinA5 IgA**	0.188	0.43	**0.454**	**0.04**
Anti-Spike IgA	0.215	0.36	0.270	0.25
Immune Complexes IgG	0.189	0.42	0.252	0.28
Immune Complexes IgA	0.110	0.64	0.092	0.70

^1,2^Correlation coefficients and significance (p-values) were calculated using Pearson test. Bold values were statistically significant.

## Discussion

Many studies have attempted to identify a critical biomarker or clinical test that would indicate a means of diagnosing or explaining the underlying pathophysiology of PASC with limited success. Deep profiling of biomarkers has suggested decreased levels of cortisol may be most predictive of PASC (18). In addition, immunologic profiles in PASC differ from controls in respect to immune cells populations and even antibody responses to non-SARS-CoV-2 pathogens (19). Our study identified correlations of IgA autoantibodies with clinical symptoms and clotting parameters, suggesting autoimmune IgA may be a possible link between coagulation and pathogenesis, which may explain the persistent respiratory symptoms of PASC.

We should acknowledge here that many of our study findings were null. For instance, there were no identifiable differences in many of the measured coagulation tests and no statistically significant differences in perfusion imaging. In addition, low levels of autoantibodies rather than high levels of autoantibodies were correlated to worse respiratory symptoms among patients with PASC. Therefore, it is entirely reasonable to conclude that this study demonstrates no evidence of autoimmunity or abnormal clotting among PASC patients. However, we would like to point out a few interesting observations from our data.

Regarding our findings from SPECT-CT imaging, it had been suggested in case reports that this type of nuclear medicine imaging might be an appropriate modality for detecting ongoing lung injury among PASC patients experiencing persistent respiratory symptoms (3, 4). However, our study outcomes suggest that such test results are not prevalent among the majority of PASC patients. In fact, only a quarter of the patients examined had abnormal results, suggesting that this approach lacks the necessary sensitivity. This lack of sensitivity may be driven by deficits that are only identifiable at smaller resolution than SPECT-CT is able to provide, or the microvascular perfusion deficits may be transient, therefore, may not be identified on a single scan of a PASC patient if they are indeed present. Furthermore, we noted that a couple of healthy post-COVID-19 controls also had identifiable perfusion deficits on SPECT-CT, which suggests that these findings are not specific to PASC. Given that these controls did not have any active respiratory symptoms, it may be that these perfusion deficits are artifactual, not clinically significant, or may be transient, especially given that one of the controls had substantial perfusion deficits, arguably worse than any of PASC cases (20).

Conversely, even if these perfusion deficits do not hold clinical significance or possibly resolve spontaneously, the process producing these deficits may have other consequences. For instance, there have been several studies demonstrating that there is a two to three fold increase in thromboembolic events such as pulmonary embolisms, deep venous thrombosis, ischemic stroke, along with other cardiovascular events in PASC patients (21). Our study found that these microvascular perfusion deficits among PASC patients may be 2.5 times higher than controls, although the small sample size precludes any definitive estimates of relative risk.

We also performed TEG profiles of cases and controls, a test that needs to be performed within hours of blood draw and quantitatively measures the ability of whole blood to form a clot. Given that there were no substantial differences in TEG results between both groups suggests that the clotting irregularities observed in PASC patients are unlikely to be caused by factors that would be required to form an *in vitro* clot (22). However, there are aspects of clotting that are not components of blood, for instance, vessel injury, blood flow characteristics, and local vessel wall biology are critical *in vivo* determinants that can increase a patient’s propensity to clot (23).

Previous studies in acute COVID-19 have observed an increase in fibrinogen and markers of tissue damage in the circulation in severe cases (24). Our study did find a statistically significant difference in thrombin time between PASC cases and healthy post-COVID-19 control participants. While the difference was small in terms of effect size, a prolonged thrombin time would be indicative of low levels of fibrinogen, impairment of fibrin formation, and/or a thrombin inhibitory effect (25). In our study, thrombin time was strongly associated with fibrinogen levels in PASC patients, but not among controls. These results may suggest that the extended thrombin time is caused by a decrease in fibrinogen levels in patients, paradoxically implying a decreased propensity to clot.

However, the balance of clotting factors requires a delicate equilibrium, raising the possibility that an *in vivo* factor not present in the blood could be enhancing clot formation. This could trigger a readjustment of blood factors, resulting in a slightly prolonged thrombin time. The *in vivo* factor, distinct from blood components, might be linked to vascular injury or a protein within the blood vessel wall that contributes to fibrin formation or fibrinolysis (26). Therefore, there could be a *in vivo* process that is contributing increased thrombosis, which would might need to be counterbalanced by peripheral circulating factors that degrade clot. In addition, there are some clinical entities where the measured levels of factors involved in the pathogenesis of disease are paradoxically low. For instance, in the clinical entity known as thrombotic thrombocytopenic purpura (TTP), clinical testing demonstrates low levels of platelets; however, this is due to the aggregation of activated platelets into clots, which lead to thrombotic events.

Our findings among PASC patients also show reduced levels of circulating antibodies targeting lung-related antigens. These findings are not likely to be a consequence of generalized lower IgA levels, since there was no significant difference in the total levels of circulating IgA between PASC patients and controls in our cohort. In this regard, our results are different from another study that observed lower levels of circulating IgA antibodies in PASC patients (27). In our results, we not only found that low total plasma IgA and anti-endothelial IgA levels were strongly correlated with clinical respiratory symptoms, but also correlated with low fibrinogen levels, low fibrin-related clot strength and low cell-free DNA in plasma. The reason for these findings is unclear but should be further explored in a larger sample of patients to confirm whether this pattern holds true among other patients with PASC. Overall, our investigations lead us to wonder whether the peripheral blood samples are not providing the whole picture of what is driving the pathophysiology of PASC. Future studies would be needed to understand the tissue level dysfunction that causes ongoing respiratory symptoms years after an initial SARS-CoV-2 infection.

## Conclusions

In our study of PASC patients with persistent respiratory symptoms, we found that IgA antibodies correlate inversely with clinical symptoms and directly with clotting parameters, suggesting a possible link between autoimmunity and coagulation, but in a unexpected direction as lower levels of IgA predicted more respiratory symptoms among PASC cases. These findings require further validation but highlight the need to study the relationship of IgA antibodies to clotting abnormalities or other disease mechanisms in PASC. Overall, it can be challenging to determine whether autoimmunity is present as the presence of autoantibodies is not sufficient for making such a determination, and more advanced immunologic studies may be required to further explain our findings. In addition, our study identified that SPECT-CT lacks the necessary diagnostic precision to differentiate respiratory PASC patients from healthy controls. If microvascular perfusion deficits are indeed present among PASC patients, it may be that alternative diagnostic approaches are required or serial testing if these perfusion deficits are transient.

### Limitations

We want to strongly caution that the number of study participants in our study was small. Therefore, the findings of our study may be artifactual due to small sample sizes or could be otherwise spurious due to the way that our assays were performed. These results should be investigated in a larger sample of patients. It is also important to note that we studied a cohort of PASC patients who did not require hospitalization during their acute COVID-19 phase and did not have abnormal pulmonary function tests or detectable abnormalities on prior CT scans. Consequently, it is possible that we inadvertently favored the inclusion of patients less likely to manifest perfusion deficit abnormalities. Finally, it is possible that these correlations identified in our study may be caused by some other process, especially given that non-specific inflammation can also cause changes in a number of biomarkers (28).

## Data Availability

The original contributions presented in the study are included in the article/**Supplementary Material**. Further inquiries can be directed to the corresponding author.
