# Supplementary material for: IgA autoimmunity and coagulation among post-acute sequelae of SARS-CoV-2 infection (PASC) patients with persistent respiratory symptoms: a case-control study
Source: Front Immunol. 2025 May 30;16:1589559. doi: 10.3389/fimmu.2025.1589559 (PMC12162584; doi:10.3389/fimmu.2025.1589559)
Supplement: Supplementary file 1 [file DataSheet1.docx]

**Supplemental Methods:** More detailed descriptions of assays.

*Enzyme-Linked Immunoassay (ELISA)*

Patient plasma samples were obtained and stored at -80°C during ongoing recruitment of study participants. Plasma samples were then defrosted for use in ELISA for determination of total levels of IgG and IgA, and for reactivity against anti-SARS-CoV-2 spike protein, two lung cell lines (human pulmonary microvascular endothelial cells - HPMEC[1] and human lung adenocarcinoma epithelial cells A549 (ATCC)), DNA, Annexin A2 and A5. Total IgG and IgA levels were measured using ELISA kits (Invitrogen) following manufacturer's’ instructions. To determine reactivity against SARS-CoV-2 virus, an in-house ELISA with the SARS-CoV-2 spike protein was performed with some modifications. Briefly, Immulon 2HB 96-well ELISA plates (Thermo Fisher Scientific) were coated at 4°C overnight with 100µL of recombinant COVID-19 S (RBD) protein (Creative Biomart, nCoVS-125V) in phosphate-buffered saline (PBS) at 5 µg/mL. Plates were washed 3 times with 250 µL of PBS 0.1% Tween-20 (PBST) and blocked for 2h at room temperature with 200µL of 1.5% non-fat dry milk in PBST. Plates were washed again 3 times and plasma samples (1:50 dilution in 0.5% non-fat dry milk in PBST) and incubated for 2h30min at room temperature. Plates were washed 3 more times and incubated with a polyclonal goat anti-human IgG-HRP or IgA-HRP (Invitrogen) diluted 1:500 in 0.5% non-fat dry milk in PBST for 1 hour at room temperature. Plates were washed 3 more times and developed with TMB substracte (BD Biosciences).

For cell lines reactivity plates were coated with 100µL of cell lysates at 110 µg/mL (HPMEC) or 130µg/mL (A549) of protein in ethanol and incubated at 4°C overnight. To measure anti-DNA antibodies, plates were coated with calf thymus DNA (predominantly double stranded, D4522; Sigma-Aldrich) at 10 μg/mL in PBS. Plates were allowed to evaporate at room temperature until completely dry, washed 3 times with 200µL of PBS 0.05% Tween-20 and then blocked for 1h at 37°C with 100µL of PBS 3% bovine serum albumin (BSA) blocking buffer. Plasma samples were diluted at 1:100 in blocking buffer and incubated duplicates for 2h at 37°C. Plates were washed again 3 times, incubated with a polyclonal goat anti-human IgG-HRP or IgA-HRP (Invitrogen) diluted 1:500 for 1 hour at 37°C. Plates were washed 4 more times and developed with TMB substracte (BD Biosciences).

Anti-Annexin A2 and anti-Annexin A5 antibodies were measured by coating plates with 10 µg/mL of human recombinant Annexin A2 or A5 produced in HEK293 cells (Ray Biotech) in PBS overnight at 4ºC. After 3 washes with 0.05% Tween-20 in PBS, the plates were blocked with 0.3% gelatin in PBS at 37ºC for 3 hours. The ELISA plates were then washed 3 times, and plasma samples (1:100 dilution) in 0.1% gelatin in PBS were added to the corresponding wells for 2 hours at 37ºC. After washing, bound antibody was detected as mentioned previously.

To determine Immune Complexes, plates were coated with C1q polyclonal antibody (1μg/ml in carbonate-bicarbonate buffer; Sigma-Aldrich) and the same protocol as for the lung cell lines was followed with the following modifications: plates were blocked for 1h30min with PBS 0.1% BSA, plasma and secondary antibody were diluted 1:80 and 1:5,000, respectively. All steps were performed at room temperature. The reactions were stopped using stop solution (Biolegend) and absorbance read in a microplate reader (VICTOR Nivo Multilabel Reader, PerkinElmer, Waltham, Massachusetts) at 450 nm. To calculate relative units (RU) the mean OD at 450nm from duplicate wells was compared with a reference positive control previously identified as high responder for autoantibodies developed after malarial infection.[2]

**Supplemental Tables: Correlations Between Levels of IgG and IgA Antibody Activity with Clinical Symptoms, Coagulation Profiles, and Thromboelastography among PASC Patients versus Healthy Controls.** Pearson correlation coefficients with p-values for significance. Results with a p-value < 0.05 shaded in light gray and results with a p-value < 0.01 shaded in dark gray.

**Supplemental Figure 1:** Flow diagram of study participant recruitment.

Did not complete questionnaires

n = 1

Did not sign informed consent

n = 2

Did not complete questionnaires

n = 5

Did not have respiratory symptoms

n = 1

Completed Procedures

n = 20

Completed Questionnaires

n = 21

Potential

Cases with PASC

n = 28

PASC Cases with Persistent Respiratory Symptoms from NYU PASC Clinic

Signed Informed Consent

n = 26

Completed Procedures

n = 20

Completed Questionnaires

n = 20

Signed Informed Consent

n = 21

Healthy Controls with Prior SARS-CoV-2 Infection but no Persistent Symptoms

Potential

healthy controls

n = 21

**Supplementary Figure 2. Comparison of different Autoimmune IgG and IgA and anti-Spike Antibodies and Immune Complex (IC) Levels against Lung Endothelial and Epithelial Cell Lines among PASC Patients versus Healthy Controls.** Levels of anti-DNA, anti-Annexin-A2, anti-Annexin-A5 IgG and IgA along with IC and anti-Spike IgA antibodies. Total Plasma IgG and IgA from cases (n = 20) or controls (n = 20). RU (Relative Units). Significance assessed by unpaired student t-test *p< 0.05.


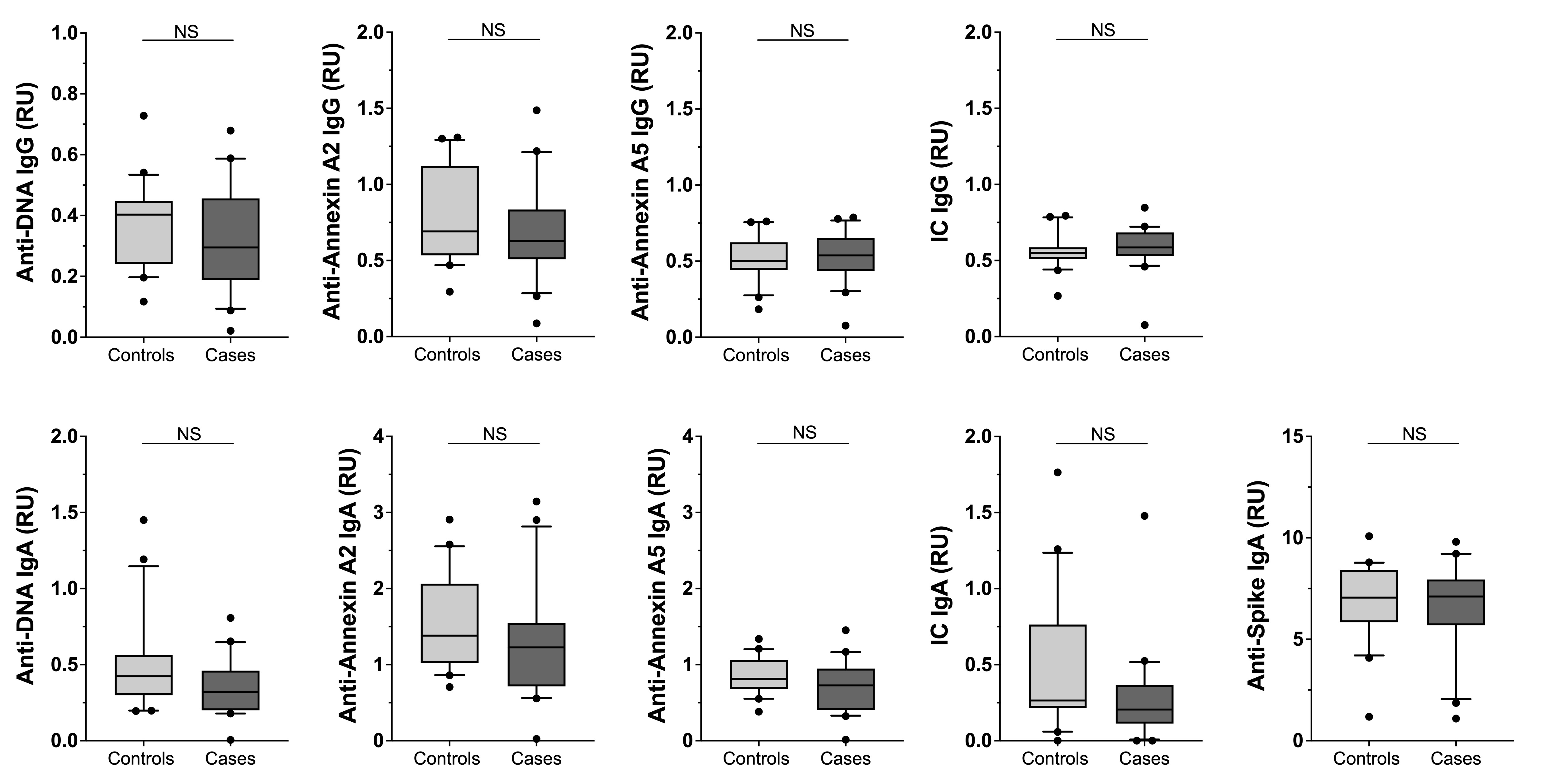


**Supplemental References**

1. Krump-Konvalinkova, V.; Bittinger, F.; Unger, R.E.; Peters, K.; Lehr, H.A.; Kirkpatrick, C.J. Generation of human pulmonary microvascular endothelial cell lines. *Lab Invest* **2001**, *81*, 1717-1727, doi:10.1038/labinvest.3780385.

2. Fernandez-Arias, C.; Rivera-Correa, J.; Gallego-Delgado, J.; Rudlaff, R.; Fernandez, C.; Roussel, C.; Gotz, A.; Gonzalez, S.; Mohanty, A.; Mohanty, S.; et al. Anti-Self Phosphatidylserine Antibodies Recognize Uninfected Erythrocytes Promoting Malarial Anemia. *Cell Host Microbe* **2016**, *19*, 194-203, doi:10.1016/j.chom.2016.01.009.
